# Psychological intervention in women victims of childhood sexual abuse: a randomized controlled clinical trial comparing EMDR psychotherapy and trauma-focused cognitive behavioral therapy

**DOI:** 10.3389/fpsyt.2024.1360388

**Published:** 2024-05-29

**Authors:** Milagros Molero-Zafra, Olga Fernández-García, María Teresa Mitjans-Lafont, Marián Pérez-Marín, María Jesús Hernández-Jiménez

**Affiliations:** ^1^ Faculty of Psychology, University of Valencia, Valencia, Spain; ^2^ Health Sciences Area, Valencian International University, Valencia, Spain

**Keywords:** childhood sexual abuse, women, EMDR, G-TEP, TF-CBT, randomized clinical trial, group psychotherapy, online psychotherapy

## Abstract

**Introduction:**

Childhood sexual abuse persists as a painful societal reality, necessitating responses from institutions and healthcare professionals to prevent and address its severe long-term consequences in victims. This study implements an intervention comprising two psychotherapeutic approaches recommended by the WHO and international clinical guidelines for addressing short-, medium-, and long-term posttraumatic symptomatology: Trauma-Focused Cognitive Behavioral Therapy (TF-CBT) and Eye Movement Desensitization and Reprocessing (EMDR). Both approaches are adapted from group formats for implementation in small online groups via Zoom.

**Methods:**

The impact of both therapeutic approaches on trauma improvement was assessed in a sample of 19 women who were victims of childhood sexual abuse through a Randomized Clinical Trial comparing EMDR Psychotherapy and Trauma-Focused Cognitive Behavioral Therapy after a baseline period. Intra and inter comparison were made using statistics appropriate to the sample.

**Results:**

Both therapeutic approaches significantly reduced symptomatology across various evaluated variables, suggesting their efficacy in improving the quality of life for these individuals. Following CBT-FT treatment, patients exhibited enhanced emotional regulation, reduced reexperiencing, and avoidance. The EMDR group, utilizing the G-TEP group protocol, significantly improved dissociation, along with other crucial clinical variables and the perception of quality of life.

**Discussion:**

Although the limitations of this study must be taken into account due to the size of the sample and the lack of long-term follow-up, the results align with existing scientific literature, underscoring the benefits of trauma-focused psychological treatments. The online group format appears promising for enhancing the accessibility of psychological treatment for these women. Furthermore, the differential outcomes of each treatment support recent research advocating for the inclusion of both approaches for individuals with trauma-related symptomatology.

**Ethics and dissemination:**

The study has been approved by the Ethics Committee of the Valencian International University (VIU) (Valencia, Spain) (Ref. CEID2021_07). The results will be submitted for publication in peer-reviewed journals and disseminated to the scientific community.

**Clinical trial registration:**

https://clinicaltrials.gov/ct2/show/NCT04813224, identifier NCT04813224.

## 1 Introduction

Sexual abuse in childhood has been and continues to be a painful reality in our society, demanding responses from institutions and health professionals concerning its prevention and the treatment of its severe long-term consequences for victims (1). Each year, tens of thousands of cases of child sexual abuse (hereinafter CSA) are reported (2). Pereda & Abad (3) indicate that CSA is one of the interpersonal victimization situations most strongly associated with psychological problems affecting all levels of an individual’s functioning throughout life, leading to significant repercussions in adulthood (4, 5) and attracting substantial research attention. In this context, the analysis of sexual abuse contributes to a profound understanding of the phenomenon, guiding practices more effectively to address it (6).

It is documented that child maltreatment (specifically, physical, sexual, and emotional abuse, as well as physical and emotional neglect) exerts a high-impact influence on the development of the child’s brain (7) and constitutes a significant risk factor for adult psychopathology (8). The effects of this traumatic experience during developmental stages often manifest in post-traumatic symptoms, post-traumatic stress disorder (PTSD), sleep problems, anxiety, depression, substance abuse, difficulties in sexual functioning, low self-esteem, and challenges in accepting one’s own body, among others (9–16). However, there is limited literature on the implementation of specific psychological treatments with randomization procedures for this population.

Recent studies have shown a specific impact on brain development due to abuse, maltreatment, and neglect during childhood, with differential effects across developmental stages. Specifically, when experienced between ages 3 and 5, there is a greater vulnerability to subsequent dissociation and PTSD; exposure to abuse and neglect between ages 10 and 11 affects the development of the amygdala; when these experiences occur between ages 11 and 13, it influences the development of the hippocampus, crucial in this developmental period; the impact at ages 14 to 16 affects the development of the prefrontal cortex (17). The affected neural processes in individuals who have experienced abuse, neglect, and child maltreatment are primarily found in the fronto-limbic networks, including the medial prefrontal cortex, orbitofrontal cortex, anterior cingulate cortex, hippocampus, and amygdala, with neglect related to changes in the development of insula activation during risk processing and abuse related to changes in fronto-parietal activation during cognitive control (18). These studies also show that a smaller volume and altered activity patterns have been observed in the ventromedial region of the prefrontal cortex in children with PTSD, suggesting the involvement of frontal lobe circuits in altered fear extinction characteristics, affecting fear conditioning and learning, with significant implications for subsequent development and consequences in adulthood.

It is important to consider all these data from recent research in neuroscience, which even indicate differential effects between neglect and abuse regarding risk processing. Neglect appears to impact more on neurodevelopment, harming the risk assessment system, while the effects of abuse on neurodevelopment involve an acceleration of the risk control system (18). Adult individuals who were victims of childhood sexual abuse have been exposed to both the experience of abuse and neglect, potentially presenting this combined impact materializing in poor emotional regulation strategies that generate difficulties at the relational level. When this combination of neglect and abuse occurs, more disruptive symptomatology in functioning has been found, associated with hypoactivation in various higher-order cortical regions, as well as in the amygdala (19). These neurocognitive functioning characteristics influence their greater vulnerability to revictimization (20, 21), representing a lifelong journey for them with a continuous stress burden related to abuse.

These pieces of evidence regarding the impact of childhood sexual abuse on adult life functioning and its implication at the neurodevelopmental level provide valuable information for effectively intervening in symptoms resulting from this neuro-emotional interference caused by trauma. Therefore, the therapies of choice for these individuals must address the impact of the event in a way that can effectively repair the post-traumatic impact, such as Eye Movement Desensitization and Reprocessing (EMDR) (22–25) and Trauma-Focused Cognitive Behavioral Therapy (TF-CBT) (26–28).

Given all this, the design of an intervention is proposed that includes these two main psychotherapeutic approaches recommended by the WHO and other international clinical guidelines for the treatment of short-, medium-, and long-term post-traumatic symptomatology (29), International Society for the Study of Trauma and Dissociation (30): Trauma-Focused Cognitive Behavioral Therapy (TF-CBT) and EMDR, both adapted from group formats to be carried out in small groups through telematic intervention via Zoom. This group and telematic format offer the possibility of reaching more people, being easily accessible, and less costly.

Trauma-Focused Cognitive Behavioral Therapy (TF-CBT) is an evidence-based treatment model designed to address post-traumatic symptoms. It can be implemented in group settings and online, offering substantial assistance to patients (children, adolescents, and young individuals) and their families in overcoming symptoms resulting from exposure to traumatic experiences (31). With extensive research supporting its efficacy, TF-CBT is now widely acknowledged as a well-established intervention for treating post-traumatic stress and associated symptoms (28, 32–38).

TF-CBT integrates components from various theoretical perspectives, including attachment theory and humanistic approaches. The influence of attachment theory is evident in the emphasis on involving a supportive caregiver, such as a partner, another family member, or a friend. This approach results in stress reduction for the victim and improved recovery. Additionally, the humanistic approach in the clinician-child relationship is valued and essential for successfully implementing TF-CBT techniques (39, 40).

The TF-CBT approach aims to: (1) explore the impact of intrafamilial childhood sexual abuse, (2) develop the victim’s sense of self-efficacy (empowerment), and (3) understand the repercussions on the behavior of minors and their social and family relationships. This therapy assists individuals in questioning and modifying dysfunctional cognitions associated with trauma (41).

Central components of TF-CBT include Psychoeducation, Relaxation, Affective Regulation (affective analysis and emotional modulation training), Cognitive Processing of trauma, Trauma Narration, Desensitization of experiences associated with trauma memories, and Enhancement of safety and future development (39). Psychoeducation about common reactions to trauma is a key feature of all TF-CBT therapies, aiming to normalize individual symptoms and justify subsequent interventions (42–44).

TF-CBT has increasingly been used in a group format, extending its application to adults who have experienced childhood sexual abuse. Authors like Deblinger, Pollio & Dorsey (45) and Bass et al. (46) assert that its application in adults and group therapy is appealing because it can be highly effective and potentially reach a larger number of individuals simultaneously. Furthermore, group TF-CBT therapy can be particularly valuable in reducing feelings of shame, isolation, and stigma experienced by victims after traumatic events (47).

As for the second chosen therapeutic modality, Eye Movement Desensitization and Reprocessing Psychotherapy (EMDR), it was developed by Shapiro (48, 49). As has already been indicated, EMDR is one of the transdiagnostic therapy models recommended in international guidelines for addressing the aftermath of traumatic life experiences. Recently, positive impacts on the neurobiology of post-traumatic stress disorder have been studied (50). This trauma-focused psychotherapeutic approach is recognized by various clinical associations and international guidelines for treating trauma, life adversities, and psychological stressors (30, 44). The World Health Organization (WHO) includes EMDR among the recommended treatments for post-traumatic stress disorder (51).

EMDR is currently a psychotherapeutic model that allows access to disturbing and traumatic life events, along with current triggers and future projected experiences. These are reprocessed with adaptive resolution (52). This approach is based on working with the processing of traumatic experiences contributing to the patient’s symptoms, with memory being the central element of therapy. It involves working with all aspects of the traumatic experience, including imagination, beliefs, affect, and bodily sensations, using dual attention stimulation through eye movements or bilateral tactile or auditory stimulation.

The standardized basic protocol consists of eight treatment phases: Phase 1 for assessment and conceptualization, Phase 2 for preparation, Phase 3 for assessing the target event, Phase 4 for processing, Phase 5 for installing an adaptive belief for the event, Phase 6 for body check, Phase 7 for closure, and Phase 8 for reevaluation. Phases 1 and 2 begin in the initial sessions and continue throughout the treatment process, allowing for the completion of case conceptualization as the selected events are worked on, using stabilization tools to manage emerging issues in the therapy process. Phases 3 to 8 are conducted with each event (traumatic memories or those related to symptoms and present situations triggering them), following a working sequence established after Phases 1 and 2 based on the diagnostic hypothesis and the individual’s specific clinical needs.

Although EMDR relies on the basic protocol described above, it can be modified and adapted to particular situations. Specific protocols have been developed for working with different clinical conditions and addressing complex traumatization and dissociation in recent years. Of particular interest for this study is the adaptation for recent traumatic events, crises or emergencies, and ongoing stress. The Recent Traumatic Event Protocol (52) was adapted and further improved by Elan Shapiro (53) to address the differences in post-traumatic symptom expression when the event has not yet consolidated in long-term memory. Subsequently, versions of this approach have been developed to enhance the effectiveness of treatment for post-traumatic stress in group interventions.

Elan Shapiro developed the Group Traumatic Events Protocol (G-TEP) to formulate a group protocol incorporating the strengths of EMDR and the AIP model, containing key elements of the individual protocol. G-TEP is an adaptation of the Recent Traumatic Event Protocol (R-TEP) for use with groups of adults, older children, and adolescents who have had recent traumatic experiences or events that have long-term life-changing consequences that are not necessarily recent. It requires a simplified and structured worksheet format suitable for quick assimilation and effective use with groups or individuals (54).

In response to a surge in requests for mental health care following natural disasters, Jarero and Artigas developed the EMDR-Group Trauma Protocol (EMDR-IGTP) (55). EMDR-IGTP has been used with children and adults worldwide, with numerous studies reporting its effectiveness in response to disasters, ongoing war trauma, ongoing geopolitical crises, war refugee displacement, workplace accidents, and severe partner violence (56). These authors have subsequently developed adaptations of these group protocols for the treatment of pathogenic memories, such as childhood sexual abuse, resulting in chronic long-term symptoms related to the impact of the accumulation of stressors over time, hindering the consolidation of such memories into long-term memory (57, 58).

The impact of trauma exposure is cumulative in nature (9). The accumulated effects of a previous trauma may be associated with more severe emotional responses to subsequent trauma (59, 60). For Stevens, Eagle, Kaminer, & Higson-Smith (61), the existing conceptualization of traumatic stress, such as PTSD and complex PTSD, may have limited utility for continuous danger and threats, given the notion that trauma exposure is localized in the past. Therefore, it does not capture the daily experiences of continuous traumatic stress without safe spaces for protection and recovery (62).

In recent years, studies have been published on the application of these protocols initially developed for recent events in populations with post-traumatic symptoms related to developmental trauma and chronic sequelae that retraumatize the individual (63). There is also evidence that women who have experienced CSA benefit from these interventions, improving their symptoms of post-traumatic stress, anxiety, and depression (63, 64).

For the current study, the G-TEP (54) and the individualized Protocol for Stabilization for Acute Stress (EMDR-PESEA) (65) were implemented. The individual application addressed potential destabilization within the group dynamic, necessitating personalized regulatory sessions for subsequent reintegration into the collective setting.

The implemented intervention is outlined as follows:

Phase 1) Client history before Session 1: An online assessment session is conducted using the LimeSurvey assessment tool, encompassing all subsequent measurement variables. This was executed on Zoom in small groups to account for potential destabilization during trauma-related questioning.

Phase 2) Preparation for the treatment of the traumatic event, including psychoeducation and regulation strategies. Resources to be used in later reprocessing phases are also prepared. The 4 Elements exercise is introduced, along with drawing a resource or symbol of a calming place, and installing positive feelings with EBL.

Phases 3-6: Conducted following the G-TEP worksheet, providing safety (safe place, past resource, desired future, timeline structure), control, order, and differentiation between past danger and present safety. The EMD strategy establishes containment limits for the present T Episode.

Phase 7) Closing the session period: A group debriefing of the experience is conducted, and some of the stabilization exercises prepared in Phase 2 (4 Elements and the container) are performed. This facilitates the patient’s transition from associative channels activated by focusing on the traumatic event to the closing phase.

Phase 8) Reevaluation: This phase takes place immediately after the group intervention. It assesses which participants may require additional individual attention and which may need further evaluation to identify the nature and scope of their symptoms.

The literature highlights several effective psychological therapies for post-traumatic stress disorder (PTSD), including various exposure therapy modalities, trauma-focused cognitive-behavioral therapy (TF-CBT), and eye movement desensitization and reprocessing (EMDR) (66–68). These therapies share central elements aimed at assisting individuals in processing traumatic memories, cognitions, and attributions, collectively known as trauma-focused psychological therapies (42, 44, 66, 69). Both TF-CBT and EMDR are recommended by the UK National Institute for Health and Care Excellence (NICE) guidelines for treating PTSD in child and adult populations, with a common form being a cycle of eight to 12 individual outpatient sessions of TF-CBT or EMDR (44).

The general objective of this study is to assess the effectiveness of trauma-focused treatments in adult women who are victims of childhood sexual abuse. Specifically, it addresses the impact of trauma related to childhood and adolescent sexual abuse in adult victims, proposing the design and implementation of two group treatment programs based on EMDR and Trauma-Focused Cognitive Behavioral Therapy. The study provides evidence of the benefits of both therapies in reducing symptoms that hinder functioning in various areas of adult life, along with a comparative analysis of their effectiveness in this population.

Based on this general objective, the following hypotheses are proposed:

### 1.1 Intrasubject hypotheses

1) Between T1 and T2, considering only the temporal progression, it is expected that there will be no change in the overall analyzed sample or in the two treatment groups analyzed separately regarding health and well-being indicators in the studied variables (life satisfaction, self-esteem, psychopathological symptoms).

2) Between T1 and/or T2 and T3, after receiving one of the two study intervention protocols (EMDR or TF-CBT), an improvement is expected in health and well-being indicators in the studied variables, both in the overall analyzed sample and in each of the two treatment groups analyzed separately. A statistically significant increase (p <0.05) is expected in life satisfaction and self-esteem, and a statistically significant reduction (p <0.05) is expected in psychopathological symptoms.

### 1.2 Intersubject hypotheses

3) At T1 and T2, it is expected that both groups (EMDR and TF-CBT) will exhibit similar scores (no statistically significant differences) in health and well-being indicators related to the studied variables (life satisfaction, self-esteem, psychopathological symptoms). This ensures that both groups are homogeneously comparable.

4) At T3, differences are expected based on the type of treatment received, either EMDR or TF-CBT psychotherapy. It is expected that one of the two treatments will result in a significantly greater improvement in health and well-being indicators than the other treatment group (higher life satisfaction and self-esteem, lower psychopathological symptoms).

## 2 Methods

### 2.1 Design

Following the CONSORT guidelines (70), which offer evidence-based recommendations for randomized trials, a Randomized Controlled Trial (RCT) is proposed, without a control group, with participants assigned to two types of treatment: 1) TF-CBT, 2) EMDR.

Following the initial assessment (T1), participants are randomly assigned to one of the two treatment groups. Subsequently, after an interval equivalent to the duration of the intervention protocols, a re-evaluation is conducted to measure changes in participants who have not yet received treatment (T2). Following this second evaluation, participants receive the assigned treatment protocol based on the earlier randomization. Upon completion of the initial treatment, participants undergo a final reassessment (T3). This approach allows for the evaluation of both the overall benefit of applying trauma-focused psychotherapy (analyzing intrasubject changes in the overall sample between T1, T2, and T3 without distinguishing the type of treatment) and an analysis of the effect of each treatment protocol separately (conducting an intrasubject analysis, this time for each treatment condition, TF-CBT on the one hand, and EMDR on the other). Similarly, the level of effectiveness is analyzed by comparing both treatment groups (intergroup comparison after applying each type of intervention in the post-treatment assessment at T3). Evaluation time points are at 0 months (T1), after eight weeks (T2), and eight weeks after receiving treatment (T3) (**Table 1**).

**Table 1:** Procedure timeline.

T1	Initial symptomatology assessment: 1^st^ Baseline
8 weeks	Randomized distribution in the two treatment conditions: CBT - EMDR
T2	Symptomatology assessment: 2^nd^ Baseline
8 weeks	CBT *vs*. EMDR Treatment
T3	Post-treatment assessment

### 2.2 Procedure

The study protocol was submitted for approval and endorsement before its implementation. The study has been approved by the Ethics Committee (CEISH) of the Valencian International University (VIU) (Ref. CEID2021_07).

Each participant receives information about the study’s purpose and procedures and provides written consent. The psychologist specialist researcher obtains consent and assent. The data is confidential and anonymous and is used solely for the study. Numeric codes will link each participant’s identifying information. Data collected is stored in a locker at the principal investigator’s workplace, and the electronic data is password-protected on the university network computer. Any modifications to the protocol will be recorded on ClinicalTrials.gov (accessed on 16th June 2022). Clinical Trial registration number: NCT04813224. Informed consent is available at Clinical Trial registration. https://clinicaltrials.gov/ct2/show/NCT04813224.

For women’s participation in the study, connections were established with organizations, particularly the non-profit entity ACASI (Association against Childhood Sexual Abuse). ACASI offers support and psychological assistance to adults who are victims of childhood sexual abuse.

Participants are informed of the evaluation and intervention protocol, provided informed consent, and committed to confidentiality. Therapy sessions for both intervention protocols were conducted in a group and online format (via the Zoom video conferencing platform). Sessions lasted approximately 60 minutes, occurring weekly. Each therapy protocol comprised eight treatment sessions with groups ranging from a minimum of 2 to a maximum of 4 participants.

To be part of the final study sample, participants needed to complete at least 6 out of 8 treatment sessions for each intervention protocol (75% of sessions). All women in the study sample received treatment. This study did not include a control group without treatment or a waiting list because symptoms in adult victims of childhood sexual abuse tend to become chronic, and the likelihood of spontaneous recovery is low.

Regarding participant randomization, it was conducted using the Randomizer.org program before recruitment through permuted block randomization. Interested participants who met inclusion criteria (with no exclusion criteria) were assigned spaces in one of the two interventions in the order of their enrollment.

Participants are kept unaware of the two experimental conditions and are not informed about the specific procedures for implementation and assignment to each treatment group. Therefore, the details are undisclosed to the participants. However, it’s important to note that associations involved in participant recruitment, as well as therapists and members of the research ethics committee endorsing the study, are aware of the existence of the two treatment groups.

The sessions were conducted by two therapists, who were present in each session and conducted both protocols simultaneously to avoid any potential interference. Additionally, having both therapists present was beneficial in case some participants exhibited dysregulation reactions, as one therapist could then proceed to a Zoom room to assist in stabilizing the situation. Both therapists are experts in EMDR and CBT-TF models and have extensive experience in teaching and conducting clinical sessions in these treatment protocols for trauma.Sample.

As illustrated in **Table 2**, the sample consisted of 19 women who were victims of childhood sexual abuse. They ranged in age from 18 to 53 years (M = 38.42; SD = 10.34) and held Spanish nationality (89.5%), except for one American and another Portuguese. The majority had university education (57.9%), 31.6% had higher education, and 10.5% had basic education. Additionally, at the time of the intervention, 63.2% were employed. Regarding their marital status, the majority were married or in a relationship (52.6%), followed by single participants (36.8%) and separated or divorced individuals (10.5%). Thus, 57.9% lived alone with their nuclear family (partner and children), 15.8% lived alone with their family of origin (parents, siblings, etc.) or shared a residence with non-family members (friends or acquaintances), and only 5.3% lived alone or alone with their child. In terms of household income, 18.2% of participants reported income levels ranging from €45,000 to €99,999, 9.1% reported incomes between €25,500 and €44,999, another 18.2% reported incomes between €16,000 and €25,499, 18.2% reported incomes between €1,000 and €15,999, 27.3% reported incomes between €6,500 and €9,999, and 9.1% reported incomes below €6,499.

**Table 2:** Demographic characteristics of the sample.

	% (*n*) /M (SD)
Age (years)	38.42 (10.34)
Nationality	
Spanish	89.5 (17)
Portuguese	5.25 (1)
American	5.25 (1)
Sexual orientation	
Heterosexual	63.2 (12)
Homosexual	21.1 (4)
Bisexual	15.8 (3)
Educational level	
Primary Education	10.5 (2)
Higher Education	31.6 (6)
University Degree	57.9 (11)
Employment status	
Working	63.2 (12)
Not working	36.8 (7)
Marital status	
Married or in partnership	52.6 (10)
Single	36.8 (7)
Separated or divorced	10.5 (2)
Cohabitation	
With the family of origin (parents, siblings, etc.)	15.8 (3)
With the nuclear family (partner and children)	57.9 (11)
With friends or acquaintances	15.8 (3)
Alone or alone with child	10.6 (2)
Annual household income	
Between 45000€ and 99999€	15.8 (3)
Between 25500€ and 44999€	10.5 (2)
Between 16000€ and 25499€	15.8 (3)
Between 10000€ and 15999€	15.8 (3)
Between 6500€ and 9999€	31.6 (6)
Below 6499€	10.5 (2)

The inclusion criteria for sample selection were as follows: (1) being female, (2) aged 18 years or older (no limits in the maximum age for the participants), and (3) having experienced childhood sexual abuse. Additionally, the exclusion criteria were: (1) severe mental illness, indicated by extreme scores on measures such as personality dimensions (71) (assessed by the DSM-5 personality questionnaire) (72), dissociation (measured by the DES), or psychopathology indicators (measured by the SCL-90 global severity index, paranoid ideation, or psychoticism subscales); (2) problematic substance use (alcohol, cocaine, etc.) based on DSM-5 criteria; and (3) ongoing regular psychological therapy specifically addressing the traumatic experience of childhood sexual abuse (CSA).

### 2.3 The intervention

Out of the total 19 women evaluated, 8 underwent treatment using the CBT-TF protocol, while 11 received treatment through the EMDR protocol. Descriptions of both treatment protocols are available in our previously published clinical trials (73), registered under Clinical Trial number NCT04813224. Summaries of both protocols are presented in **Table 3**.

**Table 3:** Treatment protocols.

Treatment protocols	
Trauma-Focused CBT-based treatment	Trauma-Focused EMDR treatment
Adaptation of the protocol of Cohen for complex trauma. TF-CBT is an evidence-based therapeutic approach for treating traumatized patients. Generate an improvement of symptoms of PTSD, dissociative, affective, or cognitive and behavioral problems. Eight weekly one-hour online group sessions per week. Three phases of 2–3 sessions each. Phase 1: TF-CBT Coping Skills for Complex Traumas. Objectives: Establishing a trusting relationship and self-regulation skills; reinforcing safety; psychoeducation; relaxation skills, mindfulness affective and cognitive coping. Phase 2: Trauma Narration and Complicated Trauma Processing. Objectives: The development of the trauma narrative; identify and examine the impact of core beliefs; development of a hierarchy of feared stimuli and a gradual exposure schedule. Live exposure to trauma memories. Phase 3: Consolidation and completion of treatment. Objectives: After processing the trauma, share the individual progress achieved with others; follow-up sessions; ensure safety and develop appropriate relationships in real-life situations.	Adaptation of Elan Shapiro's Group Traumatic Episode Protocol for adults with childhood adverse experience and complex trauma. EMDR is an evidence-based therapeutic approach for treating traumatized patients. Generate an improvement in PTSD symptoms, dissociative, affective, or cognitive and behavioral problems. Eight weekly one-hour online group sessions per week. Eight-phase EMDR protocol. Phases 3 to 7 will be conducted during sessions 3 to 8. Phase 1: Objective: Client history before session 1. Phase 2: Objectives: Preparation for the treatment of the traumatic event, with psychoeducation and regulation strategies, calming place, and setting up positive feelings with EBL. Phases 3–6: Objectives: performed following the G-TEP worksheet. Sense of security (safe place, past resource, desired future, timeline) control structure, order, differentiation of past and present. Phase 7: Closing of the session. Objectives: A group debriefing of the experience and conducting some of the stabilization exercises. Phase 8: Objectives: Re-evaluation. After the group intervention, assess any need for individual attention.

### 2.4 Outcome measures

#### 2.4.1 *Ad hoc* registry for general sociodemographic and clinical variables

Participants will be asked for information about age, gender, nationality, marital status, coexistence, educational level, employment status, and family socioeconomic status.

#### 2.4.2 Measurement of dependent variables

**Table 4** compiles the psychological variables studied along with the corresponding measurement instruments used.

**Table 4:** Psychological Variables studied and assessment instruments used.

Variable	Assessment instruments
Post-traumatic Stress	Post-traumatic Stress Disorder Symptom Severity Scale according to the DSM-5 (EGS-R)
Emotional Regulation	Scale of Emotional Regulation Difficulties (DERS)
Dissociation	Dissociative Symptom Scale (DSS)
Psychopathology	Symptom Checklist-90-Revised (SCL-R)
Self-Esteem	Rosenberg Self-Esteem Scale (RSE)
Satisfaction with Life	Satisfaction with Life Scale (SWLS)

It is widely known that difficulties in emotion regulation are closely related to the presence of previous traumatic experiences, particularly those endured during childhood, such as sexual abuse, and this association plays a key role in explaining the development of subsequent psychiatric disorders (74). Research provides evidence that chronic exposure to stress in early life leads to molecular mechanisms affecting the neuroendocrine regulation of stress, chronic inflammation, and alteration of central neural networks (75, 76). These alterations, possibly related to epigenetic changes (77), may hinder the normal mental development necessary for healthy emotional regulation and trigger difficulties in attachment (78, 79). Indeed, a history of childhood abuse is associated with increased avoidance of negative emotions, emotional dysregulation, and psychological distress (79–84). Neuroimaging literature indicates that post-traumatic stress disorder is associated with dysfunction in brain areas (prefrontal cortex, insula, amygdala, hippocampus) involved in fear conditioning or controlling emotional response (85). This is supported by a strong association between post-traumatic stress disorder and emotional dysregulation (86). Specific difficulties in emotional regulation could exacerbate PTSD symptoms, hinder the emotional processing of trauma, and perpetuate a cycle of dysregulation (87–89).

Therefore, alongside the measurement of post-traumatic stress through the EGS-R (90), two other relevant measures are introduced: emotional regulation measured through the DERS (*Difficulties in Emotion Regulation Scale*) and dissociation measured through the DSS (*Dissociative Symptom Scale*).

Regarding the assessment of post-traumatic symptomatology, the *Posttraumatic Stress Disorder Symptom Severity Scale* according to *DSM-5 (EGS-R)* is used, which has been employed for measuring PTSD symptoms in contexts of violence and sexual assaults (90) as well as in adult victims of childhood sexual abuse (91). It is a 21-item scale based on DSM-5 diagnostic criteria, assessing the presence and severity of symptoms. The scale serves as a structured evaluation instrument in Likert format (from 0 to 3 based on the frequency and intensity of symptoms) with four domains: re-experiencing, avoidance, negative alterations in cognition and mood, and hyper-arousal, and an additional section on dissociation and dysfunctionality. The EGS-R has demonstrated adequate psychometric properties in the sample with high internal consistency (α = 0.91).

*The Difficulty in Emotion Regulation Scale (DERS)* is commonly used to assess dimensions of emotion dysregulation, including non-acceptance of emotions, interference in daily life, emotional dyscontrol, limited strategies, and difficulty with goal-directed behavior, impulse control, and emotional clarity (92, 93). Emotion regulation difficulties are common in people with PTSD. The final adapted version used in this study comprises 28 items. The DERS scale exhibits good psychometric properties with a Cronbach’s α of 0.91 for the total scale.

Dissociative symptoms have been assessed using the *Dissociative Symptom Scale (DSS)* for decades in adults who experienced childhood sexual abuse (9, 94). The DSS (95) is a self-administered scale with 28 items designed to measure dissociative symptomatology. Items are scored based on the frequency of each dissociative experience on a scale from 0 to 100, where 0 represents “never” and 100 represents “always.” The DSS has good psychometric properties, with a Cronbach’s α of 0.91 in the Spanish validation (96). The Dissociative Symptom Scale (DSS) was developed to assess moderately severe levels of depersonalization, derealization, gaps in awareness or memory, and dissociative re-experiencing that would be relevant to a wide range of clinical populations, especially those who have been victims of childhood sexual abuse and post-traumatic symptomatology. The DSS has good psychometric properties, with a Cronbach’s α of 0.95 in our study.

Additionally, considering that this study will evaluate the effectiveness of two therapeutic approaches, meaning it will examine whether the level of insight differs between trauma-focused cognitive-behavioral therapy and EMDR, it is deemed appropriate to use the *Symptom Checklist-90-R (SCL90-R*; 97–99). This instrument assesses nine specific psychopathological dimensions. Mental health professionals commonly use the SCL-90-R to assess psychological symptoms and monitor patient progress during and after treatment. Symptomatology in several areas of function is common in people who were sexually abused in childhood. Therefore, an instrument such as the SCL-90-R is useful to classify symptoms by clusters, especially if there is no clear pattern of post-traumatic stress, but there is significant symptomatology resulting from the trauma. The SCL-90-R consists of three global indices of distress: Global Severity Index, Positive Symptom Distress Index, and Positive Symptom Total. Additionally, the SCL-90-R consists of nine primary symptom dimensions: somatization, obsessive-compulsive, interpersonal sensitivity, depression, anxiety, hostility, phobic anxiety, paranoid ideation (PAR), and psychoticism. This self-administered questionnaire presents 90 items describing symptoms and requires the individual to indicate on a Likert scale between 0 (not at all) and 4 (significantly or extremely) to what extent they feel bothered by each of the symptoms described. Reliability showed a high Cronbach’s alpha for the scale (α = 0.98).

The Rosenberg Self-Esteem Scale (RSS) is the most widely used instrument to measure this element (100). An increasing number of studies suggest that self-esteem is significantly associated with the aftermath of childhood sexual abuse (101). Additionally, it is estimated that self-esteem may play a mediating role in mental health problems (102). Therefore, self-esteem is considered to serve as a dimension of positive functioning and help this clinical population manage, regulate, and minimize psychological distress, thereby generating higher levels of subjective happiness, which is beneficial for mental health (103). Hence, it has been one of the dimensions evaluated in this study. The RSS assesses the feeling of satisfaction that a person has about himself/herself. It consists of ten items focusing on general feelings of self-respect and self-acceptance. The total score ranges from 10 to 40 points, with a distinction between low (scores ≤ 25), medium (26–29), and high (≥30) self-esteem. The alpha coefficient for internal consistency is very high, α = 0.92.

Several studies reveal that individuals who experienced childhood sexual abuse not only had significantly less satisfying lives but also exhibited other symptoms, such as suicidal ideation, impaired social functioning, distorted body perception, and psychosomatic illnesses (104). Consequently, a measure of overall life satisfaction is incorporated as a variable for assessment, utilizing the *Satisfaction with Life Scale (SWLS)* (105). This 5-item scale prompts participants to express their agreement with each question on a Likert scale from 1 to 7, resulting in scores ranging from a minimum of 5 to a maximum of 35, where higher scores indicate greater life satisfaction. The SWLS is recommended for use alongside scales focusing on psychopathology or emotional well-being because it evaluates the conscious evaluative judgment of individuals about their lives based on their own criteria. The reliability analysis of the scale indicated that this Spanish version exhibits good internal consistency (α = 0.85).

### 2.5 Data analysis

The assessment instruments and the study variables collected through them will be coded and captured in databases. The results obtained from this intervention will be analyzed using the Statistical Package for the Social Sciences (SPSS version 28).

The following statistical procedures have been employed for the data analysis, considering the nature of the analyzed variables and the estimated sample size:

• Descriptive Analysis: Descriptive statistics were employed to provide a comprehensive understanding of the distribution of key variables. The data information was conveyed using frequencies (Fr) and percentages (%), with means (M) utilized to indicate central tendency and Standard Deviations (SD) obtained to highlight the degree of data dispersion.

For the remaining analyses of variables within the study sample, non-parametric hypothesis testing techniques were employed. This approach was primarily due to the limited size of each protocol group, considering the focus on a clinical sample. The following analyses have been conducted using these techniques:

- Intrasubject Analysis: Non-parametric tests to compare two or more dependent or related samples have been applied. These tests are used for before-after group designs to compare differences in results in a sample (in the total sample and, separately, in the EMDR group and the TF-CBT group) measured on the same dependent variables before and after the treatment (independent variable). The Friedman test was applied for this purpose.
- Intersubject Analysis: Non-parametric tests have been used to compare two independent or unrelated samples. These tests are used to assess differences in results between two groups (the EMDR group and the TF-CBT group) measured on the same dependent variables at the same time points (both at T1 and T2 to validate the homogeneity of both samples, ensuring their comparability, and at T3 for conducting the comparative analysis post-treatment of both psychotherapy protocols). The test applied for this purpose is the Mann-Whitney U test.
- Internal Consistency Analysis (Reliability): The Cronbach’s alpha coefficient (α) was calculated for each of the instruments used in the study to estimate their reliability. This information is provided along with an explanation of each measurement instrument.

## 3 Findings

### 3.1 Intrasubject analysis (comparison T1-T2-T3)

In this section, given the numerous analyses conducted in both the inter and intra processes of mean comparisons, only those mean contrasts resulting in statistically significant differences with a probability equal to or less than 0.05, 0.01, or a trend (probability equal to or less than 0.10) will be displayed (See **Table 5**).

**Table 5:** Variables with significant differences between pre- and post-treatment data as a function of treatment protocol.

	Variable	Friedman test	p	Dunn-Bonferroni post-test	
Treatment protocol: TF-CBT					
Post-traumatic Stress Disorder Symptom Severity Scale	Re-experiencing	7.76	0.021	2.38 (0.018) 1.75 (0.08)	T3<T1 T2<T1
	Avoidance	11.04	0.004	2.38 (0.018) 2.5 (0.012)	T3<T2 T3<T1
	Dissociative symptoms	4.73	0.094	1.75 (0.08)	T3<T1
Symptom Checklist-90-Revised	Additional Items	4.67	0.097	1.88 (0.061)	T3<T1
	Global Severity Index	4.71	0.095	2.13 (0.034)	T3<T2
	Positive Symptomatic Distress Index	4.71	0.095	2.13 (0.034)	T3<T2
Scale of Emotional Regulation Difficulties	Limited access to emotion regulation strategies	6	0.05	2.25 (0.024) -1.88 (0.061)	T3<T2 T2<T1
Treatment protocol: EDMR					
Post-traumatic Stress Disorder Symptom Severity Scale	Dissociative symptoms	5.515	0.063	2.025 (0.043)	T3<T1
Symptom Checklist-90-Revised	Somatization	4.67	0.097	2.03 (0.043)	T3<T1
	Obsessive-Compulsive	9.81	0.007	3.09 (0.002) 1.71 (0.088)	T3<T1 T2<T1
	Interpersonal sensitivity	5.69	0.058	2.24 (0.025)	T3<T1
	Depression	5.71	0.058	2.24 (0.025)	T3<T1
	Psychoticism	6.53	0.038	2.35 (0.019)	T3<T1
	Additional Items	5.42	0.067	2.24 (0.025)	T3<T1
	Global Severity Index	5.44	0.066	2.24 (0.025)	T3<T1
	Positive Symptomatic Distress Index	5.07	0.079	1.81 (0.07) 2.03 (0.043)	T3<T1 T3<T2
Dissociative Symptom Scale	Depersonalization/ Derealization	2	0.041		
Satisfaction with Life Scale	Total score	6.87	0.032	-2.5 (0.012)	T3>T1

### 3.2 CBT-TF protocol sample

Upon examining *emotional regulation*, significant differences in scores between T1 and T3 are observed in the factor that assessed *Limited Access to Emotion Regulation Strategies*. This indicates that the treatment has had an impact on improving participants in this aspect, which is an important outcome as difficulties in emotional regulation are relevant in adults who have experienced childhood abuse (106). This result aligns with the reviewed literature, indicating that the impact of trauma is associated with dysfunction in areas of the brain involved in fear conditioning or the control of emotional response functioning (107). It is also consistent with some results from the application of trauma-focused CBT, where it seems effective in reducing emotional dysregulation (108).

A trend of increased distress between T1 and T2 is also observed. This may stem from the group of women confronting their avoidant tendency regarding trauma (109, 110) while addressing aspects of it during the response to the assessment protocol and being aware that it will be addressed later.

In the *measures of Post-traumatic Stress Disorder Symptom Severity*, a trend of improvement is seen in the *Reexperiencing* subscale after trauma-focused CBT treatment. There is a significant difference between T1 and T2 (p<0.08), as well as a greater significant difference between T1 and T3 (p<0.18) after the eight treatment sessions. The trend of improvement between T1 and T2 (baseline without treatment) could be attributed to the group of women gathering for the initial evaluation in a Zoom session where they all respond to the questionnaire through LimeSurvey, facing the intrapsychic exposure to the traumatic episode in a supportive group context. This hypothesis would need to be confirmed in further studies with a more significant sample. The significant improvement in this variable after treatment aligns with findings in other research, such as that of Tichelaar et al. (1).

In the *Avoidance* scale, there is a trend of improvement between T2 and T3 (p<0.18), but a greater significant difference is observed between T1 and T3 (p<0.12). This may be because as therapy progresses, avoidant symptoms decrease. This result is very relevant as the avoidance strategy is characteristic of this population, as revealed by some studies, making it challenging to address and integrate the emotional imprint of trauma (109, 110).

The *Dissociative Symptoms* subscale displays a positive trend in response to the treatment, revealing significant differences between T1 and T3 (p<0.08), with no observable change between T1 and T2. This outcome is consistent with findings in limited studies on this topic (1). While extensive evidence supports this therapy in the child population, additional research is warranted in the adult population.

In the evaluation of *psychopathological symptoms through the SCL-90*, improvement is observed in aspects evaluating additional items: *Poor appetite, Sleep problems, Thoughts about death or dying, Overeating, Waking up very early, Restless sleep, and Feelings of guilt*. A significant difference is observed (p<0.06) between T1 and T3, possibly explained by the positive effect of treatment, as there are no significant differences at baseline. The value of these items, even though they are not central to psychopathological severity, represents a significant clinical change.

In the *Global Severity Index*, a good indicator of the current level of distress severity that combines the number of symptoms recognized as present with the intensity of perceived distress, there is also a clear significant improvement due to the treatment effect between T2 and T3 (p<0.03).

In the index evaluating response style, indicating whether a person tends to exaggerate or minimize the distress they are experiencing, the *Positive Symptom Distress Index*, there is again a significant improvement due to the treatment effect between T2 and T3 (p<0.03).

These improvements noted after receiving the CBT-TF treatment protocol are clear indicators of the overall efficacy of this psychological intervention, as indicated by research such as that of Tichelaar et al. (1).

### 3.3 EMDR protocol sample

In the evaluation of *Post-traumatic Stress Disorder Symptom Severity*, after EMDR treatment, there is a trend of improvement in the dissociative symptoms subscale between T2 and T3 (p<0.06). This difference becomes statistically significant (p<0.043) when comparing T1 and T3, indicating an improvement in this variable compared to the initial assessment. This result aligAting group EMDR protocols (63).

Regarding the results in the *broad-spectrum psychopathological clinic* measured by the SCL-90, changes are observed in the following aspects:

In the *obsession and compulsion* subscale, a significant difference is noted between T1 and T2 (p<0.088), as well as a greater significant difference between T1 and T3. This suggests clear improvement after the eight treatment sessions (p<0.002). The improvement during the baseline in this symptomatic subscale could be attributed to the group of women confronting their avoidant tendency regarding trauma (109, 110) when addressing aspects of it while responding to the assessment protocol and knowing that it will be addressed later. With these results, it can be concluded that the applied EMDR treatment appears to improve ruminative and compulsive symptoms significantly. Although more research is needed, this result is in line with a systematic review conducted in 2021 on the improvement of obsessive symptomatology with EMDR treatment (111).

The items measuring *somatization* show a trend toward significance (p<0.097), with an improvement in these symptoms throughout the treatment from T1 to T3. Notably, this variable has no significant differences between T1 and T2, indicating that the change becomes evident after treatment administration in this specific subscale. Based on these findings, it can be inferred that the implemented EMDR treatment appears to alleviate somatic symptomatology significantly. This is particularly relevant as somatization is linked to childhood sexual abuse in both men and women (112).

Concerning *interpersonal sensitivity*, a significant difference is observed (p<0.05) between T1 and T3, possibly explained by the positive effect of treatment, as there are no significant differences at baseline. Here again, relevant symptomatology for this population is significantly improved after treatment, contributing to self-confidence and trust in oneself and others.

In the domain of *Depression*, a notable difference is noted (p<0.05) between T1 and T3, possibly explained by the positive effect of treatment, with no significant differences at baseline. Depressive symptomatology is highly present in this population (113, 114), and it has significantly decreased after EMDR treatment, in line with the early research on EMDR with this population (115), and more recent studies (63).

For aspects related to *Psychoticism*, a significant difference (p<0.01) is observed between T1 and T3, potentially attributed to the positive impact of treatment, with no significant differences at baseline. The noteworthy decrease in scores on this scale, encompassing items related to guilt, perception of sex, and feelings of being different, is highly positive. This reduction could indicate a trend supported by other studies: EMDR may lead to a decline in psychotic symptoms and the risk of crises in patients (116). In a similar vein, Valiente et al. (23) have found preliminary evidence suggesting that EMDR therapy improves both psychotic and affective symptoms.

In the additional *items referring to poor appetite, sleep problems, overeating, thoughts about death, feelings of guilt, and restless sleep*, a significant difference is also observed (p<0.05) between T1 and T3, possibly explained by the positive effect of treatment, as there are no significant differences at baseline. Therefore, their improvement also represents a significant clinical change.

Regarding the *Global Severity Index*, providing information on the current level of distress severity, a significant difference (p<0.05) is observed between T1 and T3, possibly explained by the positive effect of treatment, with no significant differences at baseline.

On the other hand, the *Positive Symptom Distress Index*, a measure that indicates whether a person tends to exaggerate or minimize the distress they are experiencing, shows a trend of improvement both between T2 and T3 and between T1 and T3 (p<0.10), with no significant differences at baseline, and a reduction in symptom presence after treatment.

These last three subscales related to the overall impact of symptomatology show a significant improvement after EMDR treatment, an improvement that is also seen in the CBT-TF group, in line with the overall improvement that both treatments seem to produce in this population (1).

In the context of the *Depersonalization-Derealization* clinic, significant differences (p<0.05) are observed between T1 and T3. This positive outcome, signifying a notable reduction in dissociative symptoms like depersonalization and derealization, is well-documented in earlier studies on EMDR in this population (115) and more recent research (63), as observed in our sample.

Finally, in terms of *life satisfaction*, differences are found between T1 and T3. Consistent with the outcomes observed in the previous symptoms, EMDR significantly improves life satisfaction, which is an important indicator of overall improvement.

These findings are in line with other studies indicating positive outcomes in women who are victims of CSA with both EMDR and TF-CBT treatment, yet with a more substantial reduction in PTSD symptoms in the EMDR groups (117).

### 3.4 Total sample - analysis of the effect of receiving trauma-focused treatment

A thorough intrasubject assessment has been undertaken, concurrently evaluating both treatments to observe the integrated outcomes of trauma-focused intervention (See **Table 6**). Previous analyses have already demonstrated the effectiveness of both treatments, resulting in significant changes that contribute to improving these women’s quality of life and mental health.

**Table 6:** Variables with significant differences between pre- and post-treatment data.

	Variable	Friedman test	p	Dunn-Bonferroni post-test	
Post-traumatic Stress Disorder Symptom Severity Scale	Avoidance	5.92	0.052	1.78 (0.074)	T3<T2
	Negative alterations in cognition and mood	5.25	0.073	2.11 (0.035)	T3<T1
	Dissociative symptoms	10.15	0.006	1.7 (0.089) 2.68 (0.007)	T3<T2 T3<T1
	Total score	5.62	0.06	2.27 (0.023)	T3<T1
Symptom Checklist-90-Revised	Obsessive-Compulsive	8.08	0.018	1.99 (0.052) 2.68 (0.007)	T3<T2 T3<T1
	Interpersonal sensitivity	7.37	0.025	2.51 (0.012)	T3<T1
	Depression	5.44	0.066	1.78 (0.074) 2.11 (0.035)	T3<T2 T3<T1
	Anxiety	4.76	0.093	2.11 (0.035)	T3<T2
	Psychoticism	7.31	0.026	1.87 (0.062) 2.27 (0.023)	T3<T2 T3<T1
	Additional Items	9.88	0.007	1.95 (0.052) 2.92 (0.004)	T3<T2 T3<T1
	Global Severity Index	8.22	0.016	2.27 (0.023) 2.59 (0.009)	T3<T1 T3<T2
	Positive Symptomatic Distress Index	9.08	0.011	1.95 (0.052) 2.92 (0.004)	T3<T1 T3<T2
Scale of Emotional Regulation Difficulties	Limited access to emotion regulation strategies	6	0.05	2.19 (0.029)	T3<T2
Dissociative Symptom Scale	Absorption	6.04	0.049	2.1 (0.036) 2.01 (0.045)	T3<T1 T2<T1
	Depersonalization/Derealization	8	0.015		

Regarding *Post-traumatic Stress Disorder Symptom Severity*, measured through the EGS scale:

There is a trend of improvement after treatment (p<0.07) between T2 and T3 in the *behavioral/cognitive avoidance* scale, with no significant differences between baseline times (T1 and T2). This result is consistent with previous research findings and is relevant since it is a type of symptomatology that is difficult to reduce, as patients have been regulating coping with this traumatic episode using dissociative and avoidant strategies for years (109, 110).

For the *“Negative Cognitive Alterations/Negative Mood”* subscale, a trend of improvement after treatment is observed (p<0.07) between T1 and T3, with no significant differences between baseline times (T1 and T2).

The same trend of improvement (p<0.06) is found for the total score on this scale of post-traumatic symptoms, both between T1 and T3 and between T2 and T3.

Beyond these trends, significant differences are found in *dissociative symptomatology* measured by this questionnaire (p<0.06), with this difference occurring in the dissociative symptoms subscale between T1 and T3.

In the context of the *broad spectrum psychopathological clinic*, it should be noted that:

Concerning the *Obsession and Compulsion* clinic, there is a significant difference in these symptoms (p<0.018) between T3 and T1 and between T3 and T2. No significant differences are observed in the baseline results.

Regarding *Interpersonal Sensitivity*, there is a significant difference in these symptoms (p<0.025) between T3 and T1. No significant differences are observed in the baseline results.

When addressing aspects related to *Depression*, there is a trend of improvement with p-values of <0.066 both between T3 and T1 and between T3 and T2.

Similarly, in the *Anxiety* clinic, a trend of improvement is observed with p<0.93 between T2 and T3.

Finally, in the aspects related to the assessment of *Psychoticism*, a significant difference is observed (p<0.05) between T1 and T3 and between T2 and T3, which can be explained by the positive effect of treatment, as there are no significant differences in the baseline. As mentioned earlier, this change is positive because this scale includes items related to guilt, perception of sexuality, and feeling different.

### 3.5 Intersubject analysis

#### 3.5.1 Pre-treatment comparison between EMDR and CBT-TF protocols

Before delving into a detailed comparison of the results obtained in the study variables after applying both treatment protocols, it is essential to highlight the homogeneity of both treatment groups at T1 and T2. No significant differences were observed between the two treatment groups at these two temporal points. This indicates that concerning health and well-being indicators in the studied variables, both groups are homogeneous and can be reliably compared. The consistent baseline across groups makes the intergroup results reliable and meaningful for comparison after treatment.

#### 3.5.2 Post-treatment comparison between EMDR and CBT-TF protocols

The analyses conducted, which differentially compared post-treatment results (T3) across analyzed variables based on the applied treatment protocols, did not reveal significant differences in the post-treatment variables (see **Table 7**). This evidence supports the notion that both treatment approaches are equally beneficial for this population. This is evident both in the general sample of women who are victims of childhood sexual abuse, considering the overall treatment effect, and when separately analyzing the effects of each therapy protocol.

**Table 7:** Results of post-test (T3) differential analysis according to treatment protocol implemented.

	Variable	Mann–Whitney U test	p
Post-traumatic Stress Disorder Symptom Severity Scale	Re-experiencing	54	0.442
	Avoidance	59	0.238
	Negative alterations in cognition and mood	55	0.395
	Hyper-arousal	40	0.778
	Dissociative symptoms	41	0.84
	Dysfunctionality	48	0.778
	Total score	53	0.492
Symptom Checklist-90-Revised	Somatization	49.5	0.657
	Obsessive-Compulsive	43.5	0.968
	Interpersonal sensitivity	44	1
	Depression	49.5	0.657
	Anxiety	51.5	0.545
	Hostility	52	0.545
	Phobic anxiety	38.5	0.657
	Paranoid ideation	45	1
	Psychoticism	47.5	0.778
	Additional Items	47.5	0.778
	Global Severity Index	48	0.778
	Positive Symptomatic Distress Index	41	0.657
Scale of Emotional Regulation Difficulties	Nonacceptance of emotional responses	35	0.492
	Difficulty engaging in goal-directed behavior	30	0.351
	Impulse control difficulties	52.5	0.272
	Lack of emotional awareness	32	0.492
	Limited access to emotion regulation strategies	50.5	0.6
	Lack of emotional clarity	54.5	0.395
	Emotion regulation problems (total score)	46.5	0.84
Dissociative Symptom Scale	Amnesia	30	0.272
	Absorption	36.5	0.762
	Depersonalization/Derealization	35	0.492
Rosenberg Self-Esteem Scale	Total score	49	0.717
Satisfaction with Life Scale	Total score	42.5	0.904

Consistent with existing research, such as studies by Van den Berg (118) and Mueser (119), the findings indicate that patient outcomes did not significantly differ between those who received TF-CBT or EMDR across various PTSD symptom-centered outcomes. Likewise, concerning neurophysiological changes, the study aligns with others, suggesting a comparable and beneficial psychological impact of EMDR and TF-CBT in patients with post-traumatic stress disorder. Neuroimaging data imply a similar neurophysiological substrate for clinical improvement after both EMDR and TF-CBT, involving changes affecting bilateral connectivity of the temporal pole (26).

While describing these results, it is worth noting that no differences were found, either within or between subjects, in the data related to self-esteem or in the Anxiety, Hostility, Phobic Anxiety, and Paranoid Ideation subscales of the SCL-90. These findings warrant further in-depth analysis in the future, with an increased study sample to achieve more conclusive data. The absence of treatment effects on these variables may be attributed to the small sample size of this study or the need for more extended or personalized treatments to induce the desired changes in these aspects.

## 4 Discussion

Within the broader context of this study’s general conclusions and in alignment with the overarching objective—to address the impact on adult trauma victims related to childhood sexual abuse and to assess the effectiveness of two trauma-focused treatments for these women—it is essential to note that, based on the obtained results, the first intragroup hypothesis between T1 and T2, where only the effect of time was applicable, anticipated no change in both groups in health and well-being indicators in the studied variables. This expectation aligns with Gesteira et al. (120), who assert that spontaneous recovery does not occur over time.

Concerning intragroup hypothesis 2, between T1 and/or T2 and T3, after receiving one of the two intervention protocols under study (EMDR or TF-CBT), improvement in health and well-being indicators in the studied variables was expected. This hypothesis manages to find evidence in its favor, as the results show the benefits of both trauma-focused psychotherapies in reducing symptomatology and impairing functioning in different areas of adult life. The findings make a significant contribution to research on the treatment of individuals who have experienced childhood abuse, potentially fostering the development of specific protocols for this clinical population. This aligns with studies asserting the effectiveness of trauma-focused CBT protocols, implemented over eight weeks, not only in reducing chronic posttraumatic stress disorder and depressive symptoms but also in addressing more insidious symptoms such as dissociation and emotional dysregulation (108). Moreover, recent studies by Brown (27) and Grady et al. (28) affirm the highly positive implications of TF-CBT in the treatment of childhood sexual abuse. These results are consistent with other research supporting the effectiveness of both psychotherapeutic approaches, making them preferred models for treating patients with trauma-related symptoms (121).

Regarding intergroup hypotheses, the first, at T1 and T2, expected both groups to have similar scores in health and well-being indicators in the studied variables. The evidence supports this expectation, considering that the passage of time alone does not improve well-being.

In the second intergroup hypothesis, at T3, differences based on the type of treatment received, either EMDR psychotherapy or TF-CBT, were expected. Regarding this hypothesis, both treatments have proven effective but each of them contributes to improvement in different aspects of symptomatic clinical presentation, supporting recent research advocating for the combination of these two approaches to enhance the treatment’s impact on patients who have experienced trauma. These results indicate a consistent reduction in many posttraumatic symptoms, aligning with prior research (23, 122–125) demonstrating a sustained decrease following sessions of trauma-focused CBT and EMDR treatment.

Considering the limited specific studies related to TF-CBT intervention for childhood sexual abuse in adult women, evidence supporting the efficacy of TF-CBT exists in studies focused on post-traumatic stress management in educational settings (36, 37, 126, 127), general healthcare (120), and victims of terrorist attacks (128). These authors argue that patients’ improvement is due to TF-CBT’s effectiveness, not spontaneous recovery processes.

It is also worth noting that these results support the appropriateness of online intervention (MHealth) (129), which is particularly relevant since the explosion of its use during the COVID-19 pandemic (130). Recent research indicates a positive treatment effect in online EMDR therapy using the G-TEP group protocol (131).

Several interesting results suggest avenues for further investigation in this type of intervention. For example, in the emotional regulation scale, improvements occur after applying the TF-CBT protocol, but no significant differences are observed after applying the EMDR protocol. This observation could be related to studies indicating that different trauma-focused therapy approaches improve different types of symptomatology (1). Similarly, the differential changes produced by both trauma therapy models can be interpreted. With TF-CBT, there is a trend of improvement in emotional regulation and re-experiencing, as well as significant reductions in avoidant and dissociative symptoms of post-traumatic stress. This aligns with the study by O’Doherty et al. (5), which concluded that improved mental health and PTSD symptoms after TF-CBT intervention. However, these authors acknowledge the lingering question of whether the treatment’s benefits persist over time. On the other hand, EMDR intervention stands out for the significant reduction in dissociative symptomatology, as well as significant improvements in obsessive-compulsive symptoms, interpersonal sensitivity, and psychoticism. Both therapies yield significant general improvement measures, but only EMDR shows a significant increase in life satisfaction.

Continued evidence suggests that different therapeutic approaches can yield improvements in various types of symptomatology, as indicated by Tichelaar et al. (1). Another intriguing line of interest following this study is to investigate whether the combined effect of TF-CBT and EMDR can offer greater advantages in symptom improvement and the optimal order of application. A recent meta-analysis (121) found that the best results in reducing post-traumatic symptoms were achieved with CBT approaches with exposure and EMDR therapy. They recommended incorporating both into PTSD protocols. Studies show that applying TF-CBT followed by EMDR yields significant improvements in health and well-being values. This could be explained by TF-CBT dedicating a significant phase to stabilization, which may benefit participants more (132, 133). Another study also addresses the importance of the order of both interventions (133, 134), showing evidence of improved EMDR intervention by first adding TF-CBT. Future research could delve into whether a specific order of applying both therapies (group TF-CBT and G-TEP EMDR) enhances intervention outcomes.

In conclusion, this study provides evidence of the effectiveness of trauma-focused treatments in adult women victims of childhood sexual abuse. An interesting avenue for future research would be to continue providing evidence on the efficacy and benefits of consecutively applying two protocols, allowing both types of psychotherapy to combine and enhance their efficiency and effectiveness. Additionally, it would provide insights into the potential beneficial contribution of receiving a more extended treatment (both consecutive interventions) versus a single one.

Although this study has considerable promise, it is important to consider some of the limitations that must be addressed. One such limitation is the small size of the sample. A larger number of participants would enable more rigorous analyses of demographic variables. It is also important to recognize that accessing this type of clinical population is challenging, and there is also a high experimental mortality. In this context, the initial call for participation in the study attracts a greater number of individuals who meet the specified profile. However, upon initiating contact, it is not uncommon for individuals to exhibit avoidant patterns and subsequently decline to continue. This phenomenon has been observed and will require further assessment to determine if there are strategies that can enhance the participation of this specific clinical population. Although victims of CSA exist in both men and women, it is evident that the majority of victims who seek assistance from social and health organizations specializing in the treatment of these traumatic experiences are women. It can be hypothesized that men are less visible in this context, as it is more challenging for them to share their experiences and seek help. This is reflected in the profile of the individuals who have participated in our call for pilot studies through an association of victims of CSA, who have been exclusively women.

A further limitation of this study is the lack of long-term follow-up, which would provide greater assurance regarding the interpretation of these findings. However, the reality of these women has made follow-up beyond the end of the therapeutic sessions challenging. In the future, it will be necessary to identify more effective strategies to overcome this limitation. The study’s principal strengths lie in its methodological rigor, particularly the use of a randomized controlled trial design and detailed treatment protocols for EMDR and TF-CBT. Furthermore, the innovative approach of delivering therapy in an online group format adds a unique dimension to the field. It is therefore evident that further extensive randomized clinical studies are required in order to ascertain the efficacy of these programs and to enhance the generalizability of their results. Additionally, there is a paucity of research on psychotherapy interventions for women who are victims of childhood sexual abuse, which serves to highlight the necessity for further exploration in this area.

## Data availability statement

The data that support the findings of this study are available from the corresponding author, upon reasonable request.

## Ethics statement

The studies involving humans were approved by Ethics Committee of the Valencian International University (VIU) (Valencia, Spain) (Ref. CEID2021_07). The studies were conducted in accordance with the local legislation and institutional requirements. The participants provided their written informed consent to participate in this study.

## Author contributions

MM: Writing – review & editing, Writing – original draft, Validation, Supervision, Resources, Methodology, Investigation, Formal analysis, Data curation, Conceptualization. OF: Writing – review & editing, Writing – original draft, Software, Methodology, Investigation, Formal analysis, Data curation, Conceptualization. MM: Writing – original draft. MP: Writing – review & editing, Writing – original draft, Supervision, Resources, Methodology, Investigation, Formal analysis, Data curation, Conceptualization. MH: Writing – review & editing, Writing – original draft, Resources, Project administration, Methodology, Investigation, Funding acquisition, Formal analysis, Data curation, Conceptualization.
